# *catena*-Poly[[(nitrito-κ^2^ *O*,*O*′)silver(I)]-μ-1,2-bis­[1-(pyridin-4-yl)ethyl­idene]hydrazine-κ^2^ *N*:*N*′]

**DOI:** 10.1107/S1600536811028546

**Published:** 2011-07-23

**Authors:** Alan R. Kennedy, Maurice O. Okoth, David Walsh

**Affiliations:** a Department of Pure & Applied Chemistry, University of Strathclyde, 295 Cathedral Street, Glasgow G1 1XL, Scotland; b Department of Chemistry and Biochemistry, Moi University, PO Box 1125-30100, Eldoret, Kenya

## Abstract

The asymmetric unit of the title compound, [Ag(NO_2_)(C_14_H_14_N_4_)]_*n*_, contains half of the repeating formula unit (*Z*′ = 1/2). The Ag^I^ ion lies on a twofold rotation axis. The primary structure consists of a one-dimensional coordination polymer formed by the Ag^I^ ions and the bipyridyl azine ligand in which there is an inversion center at the mid-point of the N—N bond. The nitrite anion inter­acts with the Ag^I^ ion through a chelating μ^2^ inter­action involving both O atoms. In the crystal, the coordination chains are parallel and inter­act through Ag⋯π [3.220 (2) Å] and π–π [3.489 (3) Å] inter­actions.

## Related literature

For a review of Ag(I) bipyridyl coordination behaviour, see: Khlobystov *et al.* (2001 ▶). For the synthesis and structure of related coordination polymers with azine linkers, see: Kennedy *et al.* (2005 ▶). For nitrite-containing examples, see: Chen & Mak (2005 ▶); Blake *et al.* (1999 ▶); Cingolani *et al.* (1999 ▶); Flörke *et al.* (1998 ▶); Tong *et al.* (2002 ▶).
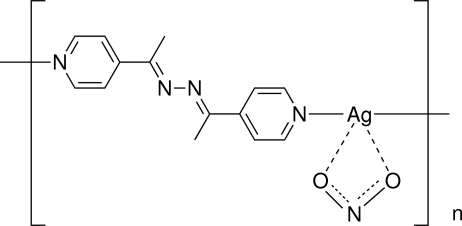


## Experimental

### 

#### Crystal data

[Ag(NO_2_)(C_14_H_14_N_4_)]
*M* *_r_* = 392.17
Monoclinic, 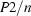

*a* = 4.8645 (2) Å
*b* = 7.3283 (2) Å
*c* = 20.7228 (6) Å
β = 93.710 (2)°
*V* = 737.19 (4) Å^3^
*Z* = 2
Mo *K*α radiation
μ = 1.38 mm^−1^
*T* = 123 K
0.30 × 0.30 × 0.28 mm

#### Data collection

Nonius Kappa CCD diffractometer
Absorption correction: multi-scan (*SORTAV*; Blessing, 1997 ▶) *T*_min_ = 0.667, *T*_max_ = 0.687
3223 measured reflections
1693 independent reflections
1487 reflections with *I* > 2σ(*I*)
*R*_int_ = 0.027

#### Refinement

*R*[*F* ^2^ > 2σ(*F* ^2^)] = 0.023
*wR*(*F* ^2^) = 0.048
*S* = 1.05
1693 reflections
102 parameters
H-atom parameters constrained
Δρ_max_ = 0.44 e Å^−3^
Δρ_min_ = −0.44 e Å^−3^

### 

Data collection: *DENZO* (Otwinowski & Minor, 1997 ▶) and *COLLECT* (Hooft, 1998 ▶); cell refinement: *DENZO* and *COLLECT*; data reduction: *DENZO*; program(s) used to solve structure: *SIR92* (Burla *et al.*, 2005 ▶); program(s) used to refine structure: *SHELXL97* (Sheldrick, 2008 ▶); molecular graphics: *ORTEP-3* (Farrugia, 1997 ▶) and *X-SEED* (Barbour, 2001 ▶); software used to prepare material for publication: *SHELXL97*.

## Supplementary Material

Crystal structure: contains datablock(s) global, I. DOI: 10.1107/S1600536811028546/lh5278sup1.cif

Structure factors: contains datablock(s) I. DOI: 10.1107/S1600536811028546/lh5278Isup2.hkl

Additional supplementary materials:  crystallographic information; 3D view; checkCIF report

## Figures and Tables

**Fig. 1.**
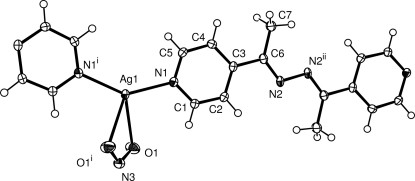
The molecular structure of (I) extended to show coordination geometry about Ag1. Non-H atoms are drawn as 50% probability displacement ellipsoids. i = 1.5 - x, y, 0.5 - z, ii = -1 - x, -y, -z.

**Fig. 2.**
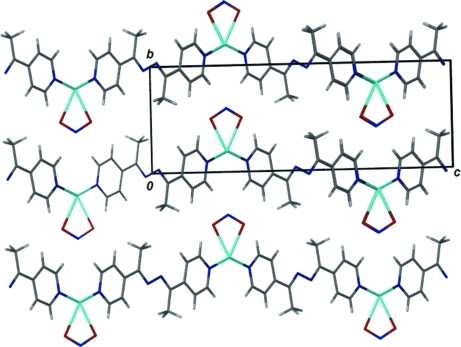
Packing diagram of (I) viewed along the crystallographic a direction.

## supplementary crystallographic information

### Comment

The formation of Ag(I) complexes of "off-axis rod" type bipyridyl ligands has attracted much interest. Partly this is due to the relative ease of crystal formation, as compared to similar systems with other metals, and partly because aggregation of the one-dimensional polymeric chains typically formed is thought to give insight into the formation of more complicated two-dimensional or three-dimensional networks (Khlobystov *et al.*, 2001). Previous work on such bipyridyl ligands containing azine chromophores showed that all displayed simple one-dimensional chains based on the coordination of two ligands to each Ag(I) centre in a *trans* manner (Kennedy *et al.*, 2005). However, the stacking of these chains is not simple - with much variation seen in the interaction types observed. With better coordinating anions Ag···anion interactions were important but Ag···Ag, Ag···solvent, Ag···azine and Ag···π contacts were also observed with little apparent systematic variation. Here we utilize the nitrite anion to limit the number of interchain Ag···anion interactions possible.

[Ag(pyC(Me)N—NC(Me)py)(NO_2_)]_n_ (I) has the expected primary chain structure with each Ag(I) centre forming two dative bonds to pyridyl fragments from two seperate ligands, see Fig 1. However, the nitrite anion also interacts with the Ag(I) centre. Its O,*O*' chelating geometry appears to be more sterically demanding than that of other anions used with such systems (*e.g.* NO_3_, ClO_4_, BF_4_ and SbF_6_) and thus the NAgN angle of 142.18 (8) ° is considerably more bent than previously seen (range 167.0 to 180 °, Kennedy *et al.*, 2005).

Whilst the observed chelating nitrite bonding mode is the commonest found in related Ag(I) complexes (see for example Blake *et al.*, 1999; Chen & Mak, 2005; Tong *et al.*, 2002) nitrite can also bridge between Ag(I) centres either through O atom coordination only (Cingolani *et al.*, 1999) or more rarely by also using the central N atom to bind (Flörke *et al.*, 1998). However, in (I) no further interactions are formed by the nitrite anion. Instead the intermolecular network expands through Ag···π interactions. Pyridyl rings lie equidistant above and below the plane of primary coordination (Ag1···C1^iii^ and Ag1···C1^iv^ are both 3.220 (2) Å, where iii is 1 + *x*, *y*, *z* and iv is 0.5 - *x*, *y*, 0.5 - *z*). Additionally the coordination chains also form π–π contacts that are within the range normally treated as significant (C3···C5^v^ = 3.489 (3) Å where v is *x* - 1, *y*, *z*) (see Fig. 2 for the crystal packing).

### Experimental

The azine ligand and complex (I) were synthesized as described in Kennedy *et al.* (2005), and crystals were grown by the solvent layering method also described therein.

### Refinement

H atoms were placed in calculated positions and refined in riding modes with C—H = 0.98 or 0.95 Å for the CH_3_ and CH groups respectively. For the methyl group *U*_iso_(H) = 1.5*U*_eq_ and for CH groups *U*_iso_(H) = 1.2*U*_eq_ of the parent C atoms.

### Crystal data

**Table:**

[Ag(NO_2_)(C_14_H_14_N_4_)]	*F*(000) = 392
*M**_r_* = 392.17	*D*_x_ = 1.767 Mg m^−^^3^
Monoclinic, *P*2/*n*	Mo *K*α radiation, λ = 0.71073 Å
Hall symbol: -P 2yac	Cell parameters from 7462 reflections
*a* = 4.8645 (2) Å	θ = 1.0–27.5°
*b* = 7.3283 (2) Å	µ = 1.38 mm^−^^1^
*c* = 20.7228 (6) Å	*T* = 123 K
β = 93.710 (2)°	Prism, orange
*V* = 737.19 (4) Å^3^	0.30 × 0.30 × 0.28 mm
*Z* = 2	

### Data collection

**Table:**

Nonius Kappa CCD diffractometer	1693 independent reflections
Radiation source: fine-focus sealed tube	1487 reflections with *I* > 2σ(*I*)
graphite	*R*_int_ = 0.027
φ and ω scans	θ_max_ = 27.5°, θ_min_ = 2.0°
Absorption correction: multi-scan (*SORTAV*; Blessing, 1997)	*h* = −6→6
*T*_min_ = 0.667, *T*_max_ = 0.687	*k* = −9→9
3223 measured reflections	*l* = −26→26

### Refinement

**Table:**

Refinement on *F*^2^	Primary atom site location: structure-invariant direct methods
Least-squares matrix: full	Secondary atom site location: difference Fourier map
*R*[*F*^2^ > 2σ(*F*^2^)] = 0.023	Hydrogen site location: inferred from neighbouring sites
*wR*(*F*^2^) = 0.048	H-atom parameters constrained
*S* = 1.05	*w* = 1/[σ^2^(*F*_o_^2^) + (0.0235*P*)^2^ + 0.063*P*] where *P* = (*F*_o_^2^ + 2*F*_c_^2^)/3
1693 reflections	(Δ/σ)_max_ < 0.001
102 parameters	Δρ_max_ = 0.44 e Å^−^^3^
0 restraints	Δρ_min_ = −0.44 e Å^−^^3^

### Special details

**Table:**

Geometry. All s.u.'s (except the s.u. in the dihedral angle between two l.s. planes) are estimated using the full covariance matrix. The cell s.u.'s are taken into account individually in the estimation of s.u.'s in distances, angles and torsion angles; correlations between s.u.'s in cell parameters are only used when they are defined by crystal symmetry. An approximate (isotropic) treatment of cell s.u.'s is used for estimating s.u.'s involving l.s. planes.
Refinement. Refinement of *F*^2^ against ALL reflections. The weighted *R*-factor *wR* and goodness of fit *S* are based on *F*^2^, conventional *R*-factors *R* are based on *F*, with *F* set to zero for negative *F*^2^. The threshold expression of *F*^2^ > 2σ(*F*^2^) is used only for calculating *R*-factors(gt) *etc*. and is not relevant to the choice of reflections for refinement. *R*-factors based on *F*^2^ are statistically about twice as large as those based on *F*, and *R*- factors based on ALL data will be even larger.

### Fractional atomic coordinates and isotropic or equivalent isotropic displacement parameters (Å^2^)

**Table:**

	*x*	*y*	*z*	*U*_iso_*/*U*_eq_	
Ag1	0.7500	0.19621 (3)	0.2500	0.01866 (8)	
O1	0.7224 (4)	0.5063 (2)	0.19907 (7)	0.0362 (4)	
N1	0.4040 (3)	0.0979 (2)	0.18357 (7)	0.0172 (4)	
N2	−0.3966 (3)	0.0334 (2)	0.02212 (7)	0.0177 (4)	
N3	0.7500	0.5978 (3)	0.2500	0.0307 (6)	
C1	0.2330 (4)	0.2146 (3)	0.15100 (9)	0.0178 (4)	
H1	0.2628	0.3418	0.1570	0.021*	
C2	0.0163 (4)	0.1591 (3)	0.10933 (9)	0.0184 (4)	
H2	−0.1003	0.2471	0.0879	0.022*	
C3	−0.0314 (4)	−0.0271 (3)	0.09871 (8)	0.0155 (4)	
C4	0.1426 (4)	−0.1477 (3)	0.13369 (9)	0.0178 (4)	
H4	0.1156	−0.2756	0.1293	0.021*	
C5	0.3544 (4)	−0.0809 (3)	0.17470 (9)	0.0180 (4)	
H5	0.4708	−0.1659	0.1978	0.022*	
C6	−0.2540 (4)	−0.0928 (3)	0.05205 (9)	0.0168 (4)	
C7	−0.2886 (5)	−0.2950 (3)	0.04252 (10)	0.0252 (5)	
H7A	−0.4465	−0.3183	0.0118	0.038*	
H7B	−0.3202	−0.3528	0.0840	0.038*	
H7C	−0.1216	−0.3458	0.0255	0.038*	

### Atomic displacement parameters (Å^2^)

**Table:**

	*U*^11^	*U*^22^	*U*^33^	*U*^12^	*U*^13^	*U*^23^
Ag1	0.01728 (12)	0.01806 (13)	0.01952 (12)	0.000	−0.00753 (8)	0.000
O1	0.0545 (11)	0.0246 (9)	0.0279 (9)	−0.0056 (8)	−0.0108 (8)	0.0019 (7)
N1	0.0160 (9)	0.0178 (9)	0.0173 (8)	0.0010 (7)	−0.0022 (7)	0.0006 (7)
N2	0.0139 (8)	0.0228 (9)	0.0156 (8)	−0.0017 (7)	−0.0040 (6)	−0.0011 (7)
N3	0.0342 (16)	0.0185 (14)	0.0373 (16)	0.000	−0.0143 (12)	0.000
C1	0.0162 (10)	0.0163 (10)	0.0204 (10)	0.0007 (8)	−0.0026 (8)	−0.0008 (8)
C2	0.0165 (10)	0.0183 (10)	0.0198 (10)	0.0023 (8)	−0.0032 (8)	0.0014 (8)
C3	0.0130 (10)	0.0204 (10)	0.0131 (9)	0.0005 (8)	0.0003 (7)	0.0004 (8)
C4	0.0194 (10)	0.0142 (10)	0.0196 (10)	−0.0010 (8)	−0.0008 (8)	−0.0004 (8)
C5	0.0168 (10)	0.0174 (11)	0.0194 (10)	0.0030 (8)	−0.0023 (8)	0.0017 (8)
C6	0.0157 (10)	0.0193 (11)	0.0153 (9)	−0.0012 (8)	0.0007 (8)	−0.0015 (8)
C7	0.0265 (11)	0.0214 (11)	0.0262 (11)	−0.0012 (9)	−0.0105 (9)	−0.0013 (9)

### Geometric parameters (Å, °)

**Table:**

Ag1—N1	2.2242 (16)	C2—C3	1.399 (3)
Ag1—N1^i^	2.2242 (16)	C2—H2	0.9500
Ag1—O1^i^	2.5058 (15)	C3—C4	1.394 (3)
Ag1—O1	2.5058 (15)	C3—C6	1.485 (3)
O1—N3	1.2501 (19)	C4—C5	1.382 (3)
N1—C5	1.343 (2)	C4—H4	0.9500
N1—C1	1.344 (2)	C5—H5	0.9500
N2—C6	1.291 (3)	C6—C7	1.503 (3)
N2—N2^ii^	1.405 (3)	C7—H7A	0.9800
N3—O1^i^	1.2501 (19)	C7—H7B	0.9800
C1—C2	1.380 (3)	C7—H7C	0.9800
C1—H1	0.9500		
			
N1—Ag1—N1^i^	142.18 (8)	C4—C3—C2	116.58 (18)
N1—Ag1—O1^i^	124.92 (6)	C4—C3—C6	121.75 (18)
N1^i^—Ag1—O1^i^	90.89 (5)	C2—C3—C6	121.67 (17)
N1—Ag1—O1	90.89 (5)	C5—C4—C3	119.94 (18)
N1^i^—Ag1—O1	124.92 (6)	C5—C4—H4	120.0
O1^i^—Ag1—O1	49.81 (7)	C3—C4—H4	120.0
N3—O1—Ag1	97.52 (13)	N1—C5—C4	123.36 (18)
C5—N1—C1	116.89 (17)	N1—C5—H5	118.3
C5—N1—Ag1	121.54 (13)	C4—C5—H5	118.3
C1—N1—Ag1	121.56 (13)	N2—C6—C3	115.29 (17)
C6—N2—N2^ii^	113.8 (2)	N2—C6—C7	126.27 (17)
O1—N3—O1^i^	115.2 (2)	C3—C6—C7	118.41 (17)
N1—C1—C2	123.34 (18)	C6—C7—H7A	109.5
N1—C1—H1	118.3	C6—C7—H7B	109.5
C2—C1—H1	118.3	H7A—C7—H7B	109.5
C1—C2—C3	119.86 (18)	C6—C7—H7C	109.5
C1—C2—H2	120.1	H7A—C7—H7C	109.5
C3—C2—H2	120.1	H7B—C7—H7C	109.5
			
N1—Ag1—O1—N3	137.41 (9)	C1—C2—C3—C4	2.2 (3)
N1^i^—Ag1—O1—N3	−55.60 (11)	C1—C2—C3—C6	−177.15 (18)
O1^i^—Ag1—O1—N3	0.0	C2—C3—C4—C5	−2.0 (3)
N1^i^—Ag1—N1—C5	0.55 (13)	C6—C3—C4—C5	177.38 (17)
O1^i^—Ag1—N1—C5	−157.90 (13)	C1—N1—C5—C4	1.0 (3)
O1—Ag1—N1—C5	163.02 (15)	Ag1—N1—C5—C4	−179.70 (14)
N1^i^—Ag1—N1—C1	179.78 (15)	C3—C4—C5—N1	0.4 (3)
O1^i^—Ag1—N1—C1	21.33 (17)	N2^ii^—N2—C6—C3	179.36 (17)
O1—Ag1—N1—C1	−17.75 (15)	N2^ii^—N2—C6—C7	1.1 (3)
Ag1—O1—N3—O1^i^	0.0	C4—C3—C6—N2	−178.81 (18)
C5—N1—C1—C2	−0.8 (3)	C2—C3—C6—N2	0.5 (3)
Ag1—N1—C1—C2	179.94 (15)	C4—C3—C6—C7	−0.4 (3)
N1—C1—C2—C3	−0.9 (3)	C2—C3—C6—C7	178.89 (18)

Symmetry codes: (i) −*x*+3/2, *y*, −*z*+1/2; (ii) −*x*−1, −*y*, −*z*.
